# Adapting malaria clinical trials infrastructure to implement COVID-19 epidemiology studies: Response to a public health emergency amid safe continuation of a research enterprise

**DOI:** 10.3389/fpubh.2022.959678

**Published:** 2022-07-26

**Authors:** John Woodford, Issaka Sagara, Alassane Dicko, Patrick E. Duffy

**Affiliations:** ^1^Laboratory of Malaria Immunology and Vaccinology, National Institute of Allergy and Infectious Diseases, Bethesda, MD, United States; ^2^Malaria Research and Training Center, University of Sciences, Techniques, and Technologies of Bamako, Bamako, Mali

**Keywords:** COVID-19, public health, malaria, pandemic response, malaria elimination program, West Africa

## Introduction

In early 2020 and in partnership with the Malian Ministry of Health, the Malaria Research and Training Center (MRTC) and the National Institute of Allergy and Infectious Diseases (NIAID) sought to assist in the response to COVID-19 in Mali. As part of the International Centers for Excellence in Research program, MRTC maintains clinical and research laboratory infrastructure in many regions of Mali to develop tools and strategies for malaria control and elimination in a decades-long collaboration with the NIAID. Multiple clinical and observational malaria studies were underway at the onset of the COVID-19 pandemic [NCT03917654 (1), NCT03952650 (2), NCT03989102 (3)]. In addition to supporting these studies, MRTC infrastructure was leveraged to address the COVID-19 pandemic, forming part of a large in-country response to swiftly redirect existing research resources (4). The rationale for the MRTC/NIAID response was 2-fold; firstly to assist in the public health response to the pandemic, and secondly to ensure the safe continuation of malaria activities in participating communities. As an added benefit, the nesting of COVID-19 epidemiology efforts within a malaria surveillance program has also positioned study sites to examine the interactions between these two diseases.

## Response from study sites

The primary components of the MRTC/NIAID response included staff education and training, institution of additional personal protective equipment (PPE) and engineering controls, adaptation of laboratory capacity to support COVID-19 serological testing, and community COVID-19 seroprevalence testing in participating communities.

Staff education and training was conducted periodically by remote meetings to ensure familiarity with emerging data, new procedures, and local guidelines. Each MRTC study site nominated a COVID-19 delegate from clinical staff with additional representation from the laboratory teams and central stores. Each delegate assessed and considered site conditions including volume of expected malaria study activities, hand hygiene infrastructure, and ventilation. Remote meetings were used to discuss site-specific improvements in participant flow for study activities, increase access to handwashing, and improve air circulation. PPE training was conducted with all clinical staff. Central stores conducted a thorough inventory and established preliminary planning for PPE conservation and reuse in discussion with site delegates.

**Figure 1:**
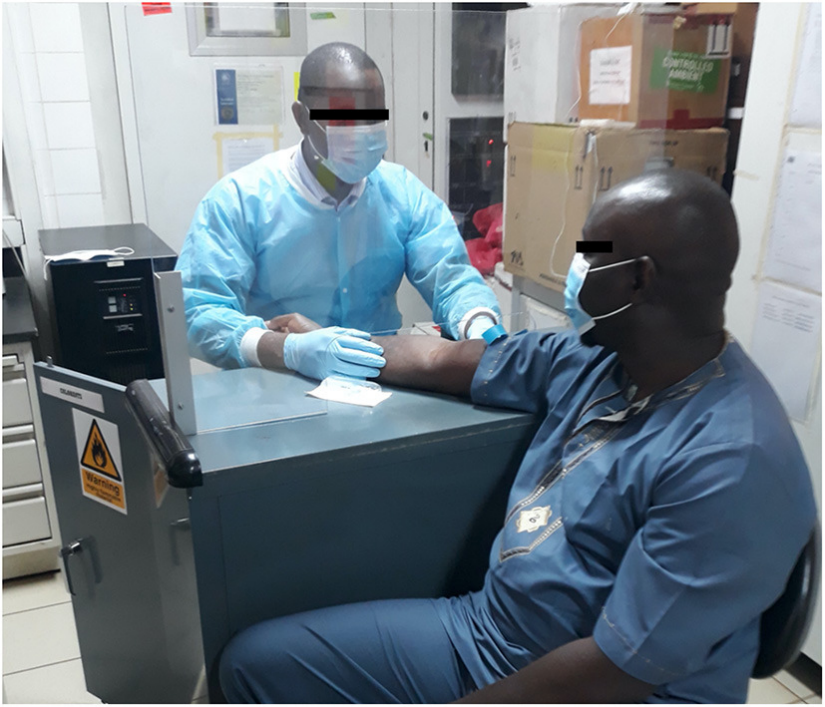
Study staff demonstrating PPE and locally manufactured barriers used at study sites in response to the COVID-19 pandemic.

Site PPE use recommendations were integrated with Ministry of Health recommendations. Local manufacturers were engaged to construct barriers to assist with physical distancing and minimize contact between participants and study staff (Figure 1). Additionally, upon request to the Ministry of Health, a supply of masks was provided for use by study participants when at the study site.

To help offset limited PCR diagnostic capacity and make use of existing local facilities and expertise, a COVID-19 ELISA developed at NIAID was transferred to MRTC laboratories (5). This assay was subsequently adapted and optimized for use in the local population (6, 7). Augmenting local PCR capabilities was not considered feasible early in the response due to challenges sourcing and shipping equipment and consumables.

Finally, in parallel to existing malaria study activities, a longitudinal cohort COVID-19 serosurvey was initiated, engaging the greater community at study sites (8). This study was primarily aimed at understanding the burden and effect of COVID-19 in study communities, where PCR diagnostics were largely unavailable. Although conducted as a standalone study, the COVID-19 serosurvey was integrated with ongoing malaria clinical trials by inviting existing participants to take part and recording existing study IDs to permit matched data analyses. Preliminary results were provided to the Ministry of Health and local authorities as a part of interval reporting to assist the public health response and the study is ongoing.

## Discussion

The well-developed MRTC/NIAID research network provided the foundation for a swift response to COVID-19, however additional factors were crucial to facilitate this relative success. Willing engagement from local health and regulatory authorities, and additional investment from the NIAID Intramural Research Program were necessary to permit these activities on top of the existing malaria program. Many collaborations were expedited or established in parallel to response activities. A multipronged approach of practical interventions adapted for local conditions helped to provide confidence to study team members, collaborators, and local authorities. Taken together, this combination of staff and participant education, engineering controls and PPE, and longitudinal community serosurveillance have helped to safely continue the malaria research program and provided valuable insights into COVID-19 epidemiology in Malian communities with limited access to public health resources. Study staff were able to continue working during this period of uncertainty and study participants were able to receive public health education and uninterrupted malaria surveillance as part of existing studies. Significantly, these activities did not include enhancing the availability of PCR diagnostics or N95 masks, which experienced global shortages in the early stages of the pandemic and continue to be in short supply in Mali. By optimizing the use of available resources and existing logistics networks, the MRTC/NIAID malaria program has helped continue malaria control activities in a largely uninterrupted fashion and established a valuable dataset to help describe the pandemic in a population that may have otherwise been overlooked. The resilience and effectiveness of the malaria program in the face of pandemic-related disruptions has highlighted the value of established long-term research networks and the importance of strong leadership from administrators and government to enable value-adding work during periods of adversity.

## Ethics statement

Written informed consent was obtained from the individual(s) for the publication of any identifiable images or data included in this article.

## Author contributions

JW drafted this manuscript. IS, AD, and PD provided revisions. All authors contributed to the article and approved the submitted version.

## Funding

JW and PD are supported by the Intramural Research Program of the National Institute of Allergy and Infectious Diseases, National Institutes of Health.

## Conflict of Interest

The authors declare that the research was conducted in the absence of any commercial or financial relationships that could be construed as a potential conflict of interest.

## Publisher's Note

All claims expressed in this article are solely those of the authors and do not necessarily represent those of their affiliated organizations, or those of the publisher, the editors and the reviewers. Any product that may be evaluated in this article, or claim that may be made by its manufacturer, is not guaranteed or endorsed by the publisher.
